# ﻿Two new species of *Colletotrichum* (Glomerellales, Glomerellaceae) causing anthracnose on *Epimediumsagittatum*

**DOI:** 10.3897/mycokeys.115.144522

**Published:** 2025-04-03

**Authors:** Kaiyun Jiang, Zhong Li, Xiangyu Zeng, Xiangsheng Chen, Shuang Liang, Wensong Zhang

**Affiliations:** 1 Key Laboratory of Agricultural Microbiology, College of Agriculture, Guizhou University, Guiyang, 550025 China Guizhou University Guiyang China

**Keywords:** *Epimediumsagittatum*, *Boninense* complex, new species, *Spaethianum* complex, pathogenicity test

## Abstract

*Epimediumsagittatum* (Sieb. et Zucc.) Maxim, a perennial herb belonging to the Berberidaceae family, is widely used in traditional Chinese medicine for its beneficial role in enhancing kidney function, strengthening bones and muscles, and dispelling wind-dampness. Clinically, it is commonly used to treat osteoporosis, rheumatism, hypertension, and cardiovascular diseases. During 2023 to 2024, a disease suspected to be anthracnose was observed to be infecting the bases of *Epimedium* seedlings in Bibo Town, Kaili City, Guizhou Province. In the fall, the disease incidence reached 90%, with severe infection resulting in total desiccation and foliage death. Tissue isolation and single-conidium methods were used to identify and isolate the pathogens, which were determined to be two anthracnose strains. Multi-locus phylogenetic analysis using ITS, *gapdh*, *act*, *tub2*, *chs-1*, *his3*, and *cal*, and morphological observations of representative isolates indicated that the two isolated fungal strains were new species belonging to the genus *Colletotrichum*, namely *Colletotrichumepimedii* and *Colletotrichumsagittati*. Pathogenicity tests, adhering to Koch’s postulates, confirmed that both fungi could infect *E.sagittatum*; *C.epimedii* exhibited a higher pathogenicity than *C.sagittati*. The present study provides valuable information regarding the prevention of *E.sagittatum* anthracnose.

## ﻿Introduction

*Epimediumsagittatum* (Sieb. et Zucc.) Maxim is a perennial medicinal plant endemic to China, primarily distributed in northern, central, and southeastern regions (He et al. 2003). Its roots and leaves are traditionally used to treat rheumatism and kidney disorders (Shen et al. 2020; Yang et al. 2020; Zheng et al. 2024). Recent studies highlight its pharmacological potential, including anti-tumour, anti-inflammatory, anti-hepatic fibrosis, and anti-osteoporosis properties. (Deng et al. 2022; Gao and Zhang 2022; Huong and Son 2023).

In October 2023, a novel leaf disease (incidence ~25%) was observed on *E.sagittatum* seedlings in Bibo Town, Guizhou Province. Initial symptoms included light brown leaf spots that expanded into greyish-white lesions with dark brown margins and yellow halos. Advanced lesions exhibited tissue thinning and saprophytic mycelial proliferation. Currently, major diseases affecting *E.sagittatum* include anthracnose, grey mould, leaf blight, and root rot (Chu et al. 2024; Hou et al. 2024a, 2024b; Zhou et al. 2024). However, research on these diseases is limited; thus, further studies are required to elucidate the diseases affecting *E.sagittatum*.

Anthracnose is a significant pathogen and endophyte widely distributed globally, which affects various hosts. Anthracnose causes substantial crop yield losses and even total crop failure annually, mainly affecting the leaves, fruits, and stems (Cannon et al. 2012; Liu et al. 2020; Stutts and Vermerris 2020). A single host plant can be infected by multiple anthrax fungi (Liu et al. 2022), whereas a single anthrax fungus can infect multiple host plants. (Damm et al. 2012; Jayawardena et al. 2021). Identification of *Colletotrichum* species is complicated by inconsistencies in morphological characteristics and host associations (Hyde et al. 2009; Jayawardena et al. 2021). However, an increasing number of *Colletotrichum* species have been identified and classified into different species complexes, such as the *Orchidearum* and *Boninense* complexes (Damm et al. 2019; Liu et al. 2022).

Therefore, accurate identification of *Colletotrichum* species is crucial for the control and prevention of anthracnose. Currently, research regarding anthracnose in *E.sagittatum* is limited. In 2024, Hou et al. (2024b) reported that *C.fructicola* caused anthracnose in *E.sagittatum* in the Henan Province. However, the specific pathogen species causing anthracnose in *E.sagittatum* in the Guizhou Province remains unknown. Thus, this study aimed to identify the pathogens responsible for *E.sagittatum* anthracnose in the Guizhou Province using a systematic classification combining morphological and multi-locus phylogenetic analyses. This research provides a theoretical basis for accurate diagnosis and effective management of the disease.

## ﻿Materials and methods

### ﻿Isolation and culturing of the pathogenic fungi

Samples of leaves exhibiting disease symptoms were collected from *E.sagittatum* in Bibo Town, Kaili City, Guizhou Province (26°30'38"N, 107°37'23"E). Pathogenic fungi were isolated from symptomatic leaves using single-spore and tissue isolation methods. If visible conidial masses were observed on the leaves, the conidia were retrieved under a microscope and transferred to sterile water to create a conidial suspension, which was then evenly spread on PDA plates in a laminar flow hood (Senanayake et al. 2020). After 24 h, the mycelium was transferred to fresh PDA plates to obtain pure cultures.

For leaves without visible conidial masses, the tissue isolation method was used to isolate the pathogenic strains. Leaf tissue pieces of approximately 0.5 × 0.5 cm were excised from the margin between healthy and diseased areas. The tissue pieces were immersed in 75% ethanol for 30 s for disinfection, followed by three washes with sterile water for a total of 30 s. The leaf pieces were then placed on sterile filter paper to dry before being transferred to PDA plates (Yao et al. 2024). All PDA plates were incubated in the dark at 28 °C for 1–2 days. Once colonies emerged, a small amount of mycelium was picked from the colony edge and transferred to fresh PDA medium for further cultivation. Colonies were purified at least twice until pure cultures were obtained. Type specimens were deposited in the Herbarium of the Department of Plant Pathology, Agricultural College, Guizhou University (HGUP). Ex-type cultures were deposited in the Culture Collection at the Department of Plant Pathology, Agriculture College, Guizhou University, P.R. China (GUCC).

### ﻿Morphological observations

The purified pathogenic fungi were inoculated onto PDA plates and incubated at 28 °C for 7 days. The colony morphology, including shape and colour, was observed and recorded according to the colour map of Rayner (1970). A small amount of mycelium from the colonies was sampled and examined using a Carl Zeiss AGAxiomo microscope to record structural details such as conidia, setae, and asci. Appressoria germination was induced by slide culture, and the conidial masses from PDA plates were transferred to sterile water to prepare a conidial suspension. A drop of this suspension was placed on a microscope slide and incubated at 28 °C for 12–24 h (Cai et al. 2009). Subsequently, the slides were observed using a Carl Zeiss AGAxiomo microscope to document appressoria shape, colour, and size. Thirty conidia and appressoria were selected randomly to measure their lengths and widths.

### ﻿DNA extraction and amplification

DNA extraction: After culturing the pathogenic fungi on PDA for 10 days, the mycelium was scraped from the plates using a sterile surgical scalpel and placed into a 2 mL centrifuge tube for storage. DNA of pathogenic fungi was extracted using a Fungal DNA (Biomiga) reagent kit and stored at -20 °C for future use.

Polymerase chain reaction (PCR) amplification was performed using the extracted DNA samples as templates. The gene sequences of the following genomic regions were amplified: rDNA internal transcribed spacer (ITS), actin (*act*), chitin synthase (*chs-1*), β-tubulin (*tub2*), glyceraldehyde-3-phosphate dehydrogenase (*gapdh*), histone H3 (*his3*), and calmodulin (*cal*). Primers used for the amplification are listed in Table 1. Each PCR reaction mixture had a total volume of 25 μL, comprising 1 μL each of forward and reverse primer, 12.5 μL of 2× PCR Master Mix, 9.5 μL of deionised water (ddH_2_O), and 1.0 μL of the DNA template (Liu 2023).

**Table 1.** Polymerase chain reaction and sequencing primers.

Target	Primer	Primer sequence (5′-3′)	Reference
ITS	ITS1	CTTGGTCATTTAGAGGAAGTAA	(Gardes and Bruns 1993; White 1994)
	ITS4	TCCTCCGCTTATTGATATGC	
*act*	ACT-512F	ATGTGCAAGGCCGGTTTCGC	(Carbone and Kohn 1999)
	ACT-783R	TACGAGTCCTTCTGGCCCAT	
*tub2*	TI	AACATGCGTGAGATTGTAAGT	(Glass and Donaldson 1995; O’Donnell and Cigelnik 1997)
	Bt2b	ACCCTCAGTGTAGTGACCCTTGGC	
*chs-1*	CHS-79F	TGGGGCAAGGATGCTTGGAAGAAG	(Carbone and Kohn 1999)
	CHS-354R	TGGAAGAACCATCTGTGAGAGTTG	
*his3*	CYLH-3F	AGGTCCACTGGTGGCAAG	(Crous et al. 2006)
	CYLH-3R	AGCTGGATGTCCTTGGACTG	
*gapdh*	GDF	GCCGTCAACGACCCCTTCATT	(Guerber et al. 2003)
	GDR	GGGTGGAGTCGTACTTGAGCATGT	
*cal*	CL1C	GAATTCAAGGAGGCCTTCTC	(Weir et al. 2012)
	CL2C	CTTCTGCATCATGAGCTGGAC	

The amplification protocol for the ITS region was as follows (Woudenberg et al. 2009): first, initial denaturation at 95 °C for 4 min; second, 35 denaturation cycles at 95 °C for 30 s, annealing at 52 °C for 30 s, and extension at 72 °C for 45 s; lastly, single extension at 72 °C for 10 min. The annealing temperatures for the other genes were as follows: 58 °C for *act*, *his3*, and *chs-1*; 55 °C for *tub2*; 59 °C for *gapdh*; and 57 °C for *cal*.

A 1.2% agarose gel was used for electrophoresis, which was stained with 0.5 g/mL ethidium bromide for 10 min. Visualisation was performed using a BIO-RAD gel imaging system. Subsequently, PCR products were sent to the Shanghai Bioengineering Company for sequencing.

### ﻿Phylogenetic analysis

Phylogenetic analysis was performed using DNA sequence data obtained from GenBank (https://www.ncbi.nlm.nih.gov/, accessed on 23 October 2024) (Tables 2, 3) and following previous publications (Liu et al. 2022; Zhang et al. 2023; Li et al. 2024). Multiple sequence alignments were performed using MAFFT (Miller et al. 2010); aligned sequences were manually adjusted using BioEdit v7.0.5 software (Rambaut 2009); using SequenceMatrix 1.8 to assemble multiple gene sequences (Vaidya et al. 2011). Phylogenetic trees were constructed using MrBayes and RAxML on the CIPRES Science Gateway V.3.3 website (https://www.phylo.org/portal2/login.action) (Miller et al. 2010; Weir et al. 2012). For the maximum likelihood (ML) analysis, RAxML-HPC2 on XSEDE v.8.2.12 was employed with a PHYLIP-formatted sequence alignment file under the GTR+GAMMA nucleotide substitution model. Branch support values were estimated through 1,000 bootstrap replicates, and the final ML tree retained branch lengths. For the Bayesian inference (BI) analysis, MrBayes v.3.2.6 was executed using a NEXUS-formatted alignment file. The optimal nucleotide substitution model was selected via MrModeltest v.2.3. Markov Chain Monte Carlo (MCMC) simulations were run with sampling every 1,000 generations. To ensure convergence, the first 25% of samples were discarded as burn-in, and a majority-rule consensus tree was generated from the remaining post-burn-in samples. Branch credibility was assessed using posterior probabilities (PP). The resulting tree files were visualised and resized using FigTree v1.4.0 (Rambaut et al. 2018) and then edited with Adobe Illustrator CS5.

**Table 2.** Sequence information for the strains used in *C.spaethianum* for multigene phylogenetic analysis. T = Type.

Species	Strain No.	Gene Bank Accession Number					
		ITS	*gapdh*	*act*	*tub2*	*chs-1*	*his3*
*C.bletillum*	CGMCC3.5117 T	JX625178	KC843506	KC843542	JX625207	MZ799322	MZ673854
*C.guizhouensis*	CGMCC3.15112 T	JX625158	KC843507	KC843536	JX625185	MZ799321	MZ673850
*C.guizhouensis*	CGMCC3.15113	JX625164	KC843508	KC843537	JX625192		
*C.incanum*	ATCC 64682 T	KC110789	KC110807	KC110825	KC110816		KC110798
*C.incanum*	CBS133485	KC110787	KC110805	KC110823	KC110814		KC110796
*C.incanum*	YYH-2	OL457651	OL439729	OL539406	OL439728	OL539407	
*C.incanum*	CBS130835	KR003337	KR003362	KR003347	KR003342	KR003357	KR003352
*C.incanum*	JZB330312	OL377888	OL471265	OL471267	OL471271	OL471269	
*C.incanum*	lL9A	KC110788	KC110806	KC110824	KC110815		KC110797
*C.incanum*	CAUCT34	KP145641	KP145573	KP145505	KP145675	KP145539	KP145607
*C.lilii*	CBS109214	GU227810	GU228202	GU227908	GU228104	GU228300	GU228006
*C.liriopes*	CBS122747	GU227805	GU228197	GU227903	GU228099	GU228295	GU228001
*C.liriopes*	CBS119444 T	GU227804	GU228196	GU227902	GU228098	GU228294	GU228000
*C.liriopes*	NN071073	MZ595908	MZ664093	MZ664206	MZ674026	MZ799326	MZ673928
*C.liriopes*	LC11287	MZ595843	MZ664092	MZ664141	MZ673964	MZ799325	MZ673863
*C.liriopes*	LC7623	MZ595842	MZ664091	MZ664140	MZ673963	MZ799324	MZ673862
*C.liriopes*	BZG101	MH291212	MH291256	MH292787		MH291234	MH292815
*C.liriopes*	DHJGF-Z5170	KC244167	KC843505	KC843543	KC244160		
*C.kingianum*	DHJGF-ZY	MW537100	MW537070	MW537088	MW537094	MW537076	MW537082
*C.kingianum*	DHJGF-P	MW537103	MW537075	MW537093	MW537099	MW537081	MW537087
*C.disporopsis*	GUCC12152	OP723106	OP784050	OP740146	OP761917		OP784175
*C.disporopsis*	GUCC12153	OP723107	OP784051	OP740147	OP761918		OP784176
*C.riograndense*	COAD928 T	KM655299	KM655298	KM655295	KM655300	KM655297	
*C.spaethianum*	CCPO34	MH020771	MH020772	MH045677	MH045678	MH020773	
*C.spaethianum*	CBS167.49 T	GU227807	GU228199	GU227905	GU228101	GU228297	GU228003
*C.spaethianum*	AJ006	KT122847	KT122856	KT122853		KT122850	MF362664
*C.spaethianum*	CLHJY4-3	MH453905	MH456883	MH456881	MH456884	MH456882	
*C.spaethianum*	CFCC57499	OP437705	OP455897	OP455891	OP455900	OP455894	
*C.tofieldiae*	CBS495.85	GU227801	GU228193	GU227899	GU228095	GU228291	GU227997
*C.tofieldiae*	CBS168.49	GU227802	GU228194	GU227900	GU228096	GU228292	GU227998
*C.verruculosum*	IMI45525 T	GU227806	GU228198	GU227904	GU228100	GU228296	GU228002
*C.iris*	LC3697*	MZ595837	MZ664090	MZ664135	MZ673958	MZ799323	MZ673856
*C.bicoloratum*	NN055229 T	MZ595899	MZ664100	MZ664197	MZ674017	MZ799332	MZ673919
*C.kingianum*	DHJGF-ML	MW537101	MW537071	MW537089	MW537095	MW537077	MW537083
*C.destructivum*	KACC 47639	OR31676	OR449456	OR449427	OR449416	OR449433	OR449494
*C.destructivum*	CBS136228	KM105207	KM105561	KM105417	KM105487	KM105277	KM105347
***C.epimedii***	**GUCC 24-0190**	**PQ555629**	**PQ650625**	**PQ650629**	**PQ650626**	**PQ650632**	**PQ650622**
***C.epimedii***	**GUCC 24-0191**	**PQ555630**	**PQ655137**	**PQ650630**	**PQ650628**	**PQ650633**	**PQ650623**
***C.epimedii***	**GUCC 24-0192**	**PQ555631**	**PQ655138**	**PQ650631**	**PQ650627**	**PQ650634**	**PQ650624**

**Table 3.** Sequence information for the strains used in *C.boninense* for multigene phylogenetic analysis. T = Type.

Species	Strain No.	Gene Bank Accession Number						
		ITS	*gapdh*	*act*	*tub2*	*chs-1*	*his3*	*cal*
*C.boninense*	CBS 123755 T	MH863323	JQ005240	JQ005501	JQ005588	JQ005327	JQ005414	JQ005674
*C.watphraense*	MFLUCC14-0123T	MF448523	MH049479	MH376384	MH351276			
*Colletotrichum* sp.	CBS 123921	JQ005163	JQ005250	JQ005511	JQ005597	JQ005337	JQ005424	JQ005684
*C.torulosum*	CBS 128544 T	JQ005164	JQ005251	JQ005512	JQ005598	JQ005338	JQ005425	JQ005685
*C.torulosum*	CBS 102667	JQ005165	JQ005252	JQ005513	JQ005599	JQ005339	JQ005426	JQ005686
*C.doitungense*	MFLUCC 14-0128 T	MF448524	MH049480	MH376385	MH351277			
*C.cymbidlicola*	IMl 347923 T	JQ005166	JQ005253	JQ005514	JQ005600	JQ005340	JQ005427	JQ005687
*C.cymbidlicola*	CBS 128543	JQ005167	JQ005254	JQ005515	JQ005601	JQ005341	JQ005428	JQ005688
*C.oncidii*	CBS 129828 T	JQ005169	JQ005256	JQ005517	JQ005603	JQ005343	JQ005430	JQ005690
*C.oncidii*	CBS 130242	JQ005170	JQ005257	JQ005518	JQ005604	JQ005344	JQ005431	JQ005691
*C.diversum*	LC11292 T	MZ595844	MZ664081	MZ664142	MZ673965	MZ799272	MZ673864	
*C.beeveri*	CBS 128527 T	JQ005171	JQ005258	JQ005519	JQ005605	JQ005345	JQ005432	JQ005692
*C.beeveri*	NN004142	MZ595881	MZ664082	MZ664179		MZ799277	MZ673901	
*C.colombiense*	CBS 129818 T	JQ005174	JQ005261	JQ005522	JQ005608	JQ005348	JQ005435	JQ005695
*C.karstii*	CBS127597 T	JQ005204	JQ005291	JQ005552	JQ005638	JQ005378	JQ005465	JQ005725
*C.karsti*	CBS 110779	JQ005198	JQ005285	JQ005546	JQ005632	JQ005372	JQ005459	JQ005719
*C.annellatum*	CBS 129826 T	JQ005222	JQ005309	JQ005570	JQ005656	JQ005396	JQ005483	JQ005743
*C.citricola*	ACCC 35478	OR240824	OR251061	OR251089	OR251096	OR251075	OR251082	OR251103
*C.citricola*	CBS 134228 T	KC293576	KC293736	KC293616	KC293656	KC293792		
*C.camelliae-japonicae*	CGMCC 38118 T	KX853165	KX893584	KX893576	KX893580	MZ799271	MZ673859	
*C.phyllanthi*	CBS 175.67 T	JQ005221	JQ005308	JQ005569	JQ005655	JQ005395	JQ005482	JQ005742
*C.petchii*	CBS 125957	JQ005226	JQ005313	JQ005574	JQ005660	JQ005400	JQ005487	JQ005747
*C.petchii*	CBS 378.94 T	JQ005223	JQ005310	JQ005571	JQ005657	JQ005397	JQ005484	JQ005744
*C.feijoicola*	CBS 144633 T	MK876413	MK876475	MK876466	MK876507			
*C.feijoicola*	CPC 34245	MK876414	K876474	MK876465	MK876506	MK876471	MK876477	
*C.limonicola*	CPC 27861	KY856471	KY856295	KY856044	KY856553		KY856387	
*C.limonicola*	CBS 142410 T	KY856472	KY856296	KY856045	KY856554	KY856213	KY856388	
*C.novaezelandiae*	CBS 130240	JQ005229	JQ005316	JQ005577	JQ005663	JQ005403	JQ005490	JQ005750
*C.parsonsiae*	CGMCC 35126	JX625181	KC843500	KC843561	JX625210			
*C.condaoense*	CBS 134299 T	MH229914	MH229920		MH229923	MH229926	MH229927	
*C.condaoense*	CBS 135989	MH229916	MH229922		MH229925			
*C.condaoense*	CBS 135823	MH229915	MH229921		MH229924			
*C.brasiliense*	CBS 128501 T	JQ005235	JQ005322	JQ005583	JQ005669	JQ005409	JQ005496	JQ005756
*C.brasiliense*	CBS 128528	JQ005234	JQ005321	JQ005582	JQ005668	JQ005408	JQ005495	JQ005755
*C.brasiliense*	TFL33.2	PP291938		PP318622	PP318621	PP318624	PP318625	PP318623
*C.hippeastri*	CBS 125376 T	JQ005231	JQ005318	JQ005579	JQ005665	JQ005405	JQ005492	JQ005752
*C.hippeastri*	CBS 125377	JQ005230	JQ005317	JQ005578	JQ005664	JQ005404	JQ005491	JQ005751
*C.hippeastri*	CBS 241.78	JQ005232	JQ005319	JQ005580	JQ005666	JQ005406	JQ005493	
*C.constrictum*	CBS 128503	JQ005237	JQ005324	JQ005585	JQ005671	JQ005411	JQ005498	JQ005758
*C.constrictum*	CBS 128504 T	JQ005238	JQ005325	JQ005586	JQ005672	JQ005412	JQ005499	JQ005759
*C.constrictum*	BXG-1	MW828148	MW855886	MW855882	MW855888	MW855884.		
*C.dacrycarpi*	CBS 130241 T	JQ005236	JQ005323	JQ005584	JQ005670	JQ005410	JQ005497	JQ005757
*C.bromeliacearum*	LC13854	MZ595833	MZ664078	MZ664131		MZ799268		
*C.bromeliacearum*	LC13855	MZ595834	MZ664079	MZ664132		MZ799269		
*C.bromeliacearum*	LC0951 T	MZ595832	MZ664077	MZ664130	MZ673956	MZ799267	MZ673843	MZ799233
*C.araujiae*	BBB:GR3504 T	OP035058	OP067659		OP067660			
*C.cliviigenum*	CBS 146825 T	MZ064415	MZ078178	MZ078143	MZ078260	MZ078161	MZ078180	
*C.chongqingense*	CS0612 T	MG602060	MG602022	MT976107	MG602044	MT976117		
*C.spicati*	CGMCC 38942 T	OL842171	OL981266	OL981240	OL981226	OL981292		
*C.celtidis*	GUCC 12014	OP723045	OP784060	OP740155	OP761926	OP730613	OP784180	
*C.chamaedorea*	NN052884		MZ664083	MZ664187	MZ674007	MZ799273	MZ673909	
*C.chamaedorea*	NN052885 T		MZ664084	MZ664188	MZ674008	MZ799274	MZ673910	
*C.catinaense*	CBS 142417 T	KY856400	KY856224	KY855971	KY856482	KY856136	KY856307	
*C.parsonsiae*	CBS 128525 T	JQ005233	JQ005320	JQ005581	JQ005667	JQ005407	JQ005494	JQ005754
*C.bromeliacearum*	LC13856	MZ595835	MZ664080	MZ664133		MZ799270		
*C.brassicicola*	CBS 101059 T	JQ005172	JQ005259	JQ005520	JQ005606	JQ005346	JQ005433	JQ005693
*C.bromeliacearum*	LC13854	MZ595833	MZ664078	MZ664131		MZ799268		
*C.palki*	CCCT 23.04 T	OR644584	OR644991	OR645097	OR645149	OR645044	OR659722	
*C.laurosilvaticum*	RGM 3086	OR644581	OR644988	OR645094	OR645146	OR645041	OR659719	
*C.laurosilvaticum*	RGM 3406	OR644582	OR644989	OR645095	OR645147	OR645042	OR659720	
*C.wuxuhaiense*	F 34	OL842173	OL981268	OL981242	OL981228	OL981294		
*C.wuxuhaiense*	YMF1.04951	OL842175	OL981270	OL981244	OL981230	OL981296		
*C.orchidophilum*	CBS 632.80	JQ948151	JQ948481	JQ949472	JQ949802	JQ948812	JQ949142.1	
*C.orchidophilum*	Clo-170	OR515649	OR566949	OR589427	OR640720			
*C.orchidophilum*	COAD 3300	MZ726565	ON512560	ON512556	ON512563	ON512557		
***C.sagittati***	**GUCC 24-0193**	**PQ555633**	**PQ664909**	**PQ655139**	**PQ655148**	**PQ655142**	**PQ655145**	**PQ655151**
***C.sagittati***	**GUCC 24-0194**	**PQ555634**	**PQ664910**	**PQ655140**	**PQ655149**	**PQ655143**	**PQ655146**	**PQ655152**
***C.sagittati***	**GUCC 24-0195**	**PQ555635**	**PQ664911**	**PQ655141**	**PQ655150**	**PQ655144**	**PQ655147**	**PQ655153**

### ﻿Pathogenicity assay

The pathogenic fungus was isolated and purified from diseased *Epimedium* plants before performing a Koch’s postulate reinoculation experiment. The strains were cultured in a 28 °C incubator for 10–25 days until conidia were produced. Using a punch, fungal cakes were harvested and transferred to conical flasks containing potato dextrose broth (PDB). These flasks were then placed on a shaker set to 220 rpm at 28 °C and cultured for 5 days to prepare a conidial suspension. The conidia concentration was determined using a hemocytometer, and the conidial suspension was adjusted to 1 × 10^6^ conidia/mL using sterile water (Guo 2023). Subsequently, this conidial suspension was sprayed onto the leaves of healthy, injured, and uninjured *Epimedium* plants for pathogenicity assessment (Than et al. 2008; Cai et al. 2009), with three replicates per treatment and spraying plants with sterile water as controls. The inoculated plants were covered with plastic bags to maintain humidity and placed in a controlled environment chamber (28 °C, 12 h light/12 h dark, 80% humidity) for observation. Regular assessments of disease development were conducted, and the results were recorded. After disease onset, the pathogen was isolated and purified from the diseased tissue.

## ﻿Results

### ﻿Field symptom observation

**Figure 1.**
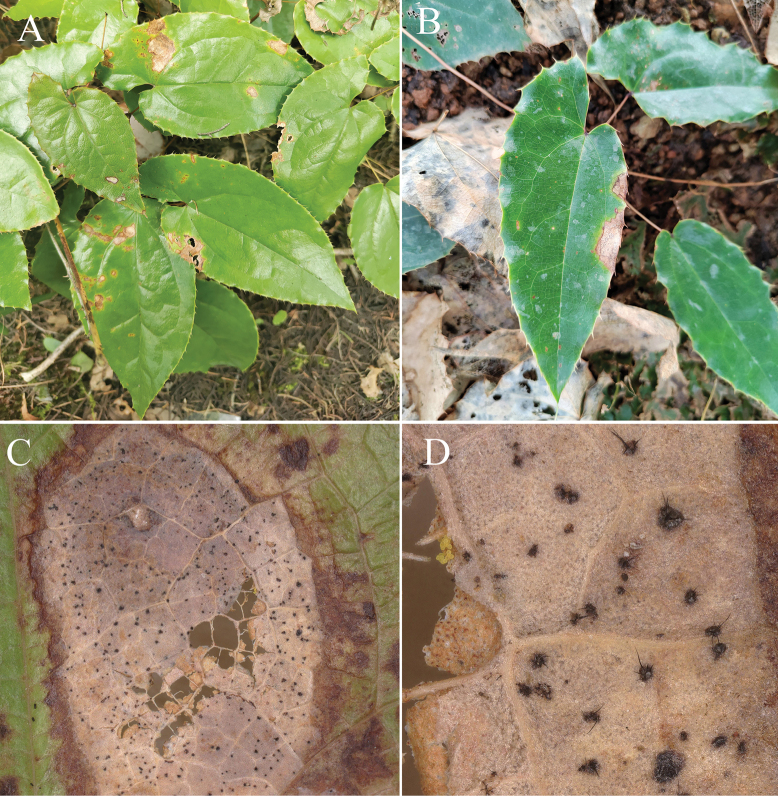
Symptoms of field anthracnose in *Epimediumsagittatum*.

The disease typically starts in April, persisting until October, with a peak incidence from June to August, when the disease incidence reaches up to 25%, and in severe cases, up to 90%. Early symptoms manifest as small light brown or brown circular spots on the middle or edges of the leaves. Gradually, these lesions expand into circular, elliptical and irregular shapes, often accompanied by irregular concentric rings. The centre of the lesions eventually turns greyish-white or grey-brown, and the margins turn dark brown and are surrounded by a yellow halo (Fig. 1A, B). In the late stages of the disease, the lesions become thin and prone to cracking, with distinct fruiting bodies; conidial heads can be observed microscopically (Fig. 1C, D).

### ﻿Pathogenicity assessment

**Figure 2.**
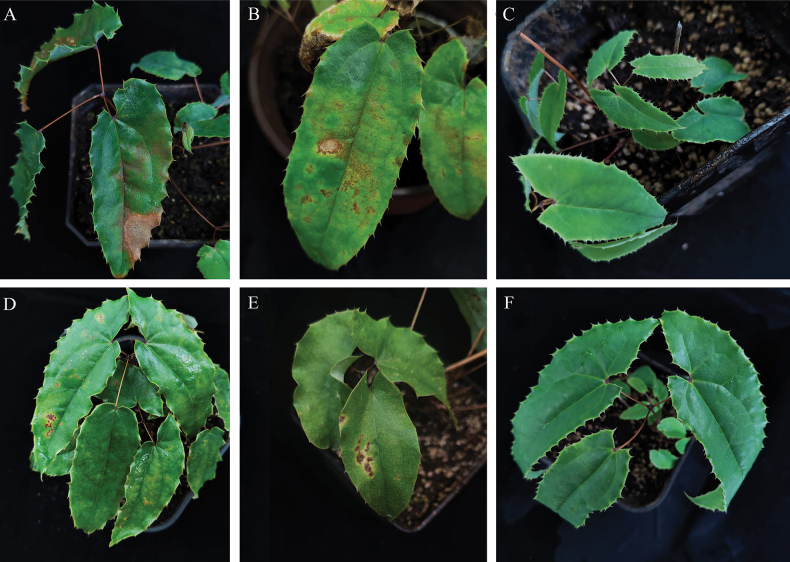
Pathogen inoculation and symptom (10 days) **A, B** symptoms resulting from inoculation with *Colletotrichumepimedii***C** control **D, E** symptoms resulting from inoculation with *Colletotrichumsagittati***F** control.

A total of 16 *C.epimedii* and 9 *C.sagittati* isolates were obtained in this study. Three representative strains from each species were selected for pathogenicity re-inoculation experiments. Symptoms began to appear four days after inoculation with *C.epimedii*, characterised by localised leaf yellowing and discolouration and accompanied by irregular brown spots. Over time, the lesions resembled the symptoms observed in the natural fields (Figs 1A, B, 2A, B). Non-treated control plants remained healthy without any symptoms (Fig. 2C). Similarly, *C.sagittati* could infect healthy plants but exhibited weaker pathogenicity, requiring wound inoculation to induce disease development. The resultant symptoms resembled field observations (Fig. 2D, E). Negative controls inoculated with sterile water remained asymptomatic (Fig. 2F).

Following pathogenicity assays, lesion margin tissues from inoculated *E.sagittatum* leaves were subjected to re-isolation of both *Colletotrichum* species. Morphological characterisation of the re-isolated pathogens revealed identical conidial dimensions and colony characteristics to those of the original isolates. These findings were consistent with initial isolation data, confirming *C.epimedii* and *C.sagittati* as the causal agents of anthracnose in *E.sagittatum*.

### ﻿Phylogenetic analysis

Twenty-five strains of *Colletotrichum*, isolated from leaves of *E.sagittatum*, were identified based on phylogenetic analyses of six or seven loci. In the phylogenetic analysis of the *C.spaethianum* species complex, a total of 2327 characters, including gaps, were identified (ITS: 538, *act*: 237, *chs-1*: 251, *gapdh*: 255, *his3*: 373 and *tub2*: 673). Similarly, the phylogenetic analysis of the *C.boninense* species complex yielded a total of 2583 characters, including gaps (ITS: 554, *act*: 254, *chs-1*: 251, *gapdh*: 242, *his3*: 375, *tub2*: 500, and *cal*: 407). The topology of Bayesian analysis of cascading datasets is almost the same as the ML consistency tree.

**Figure 3.**
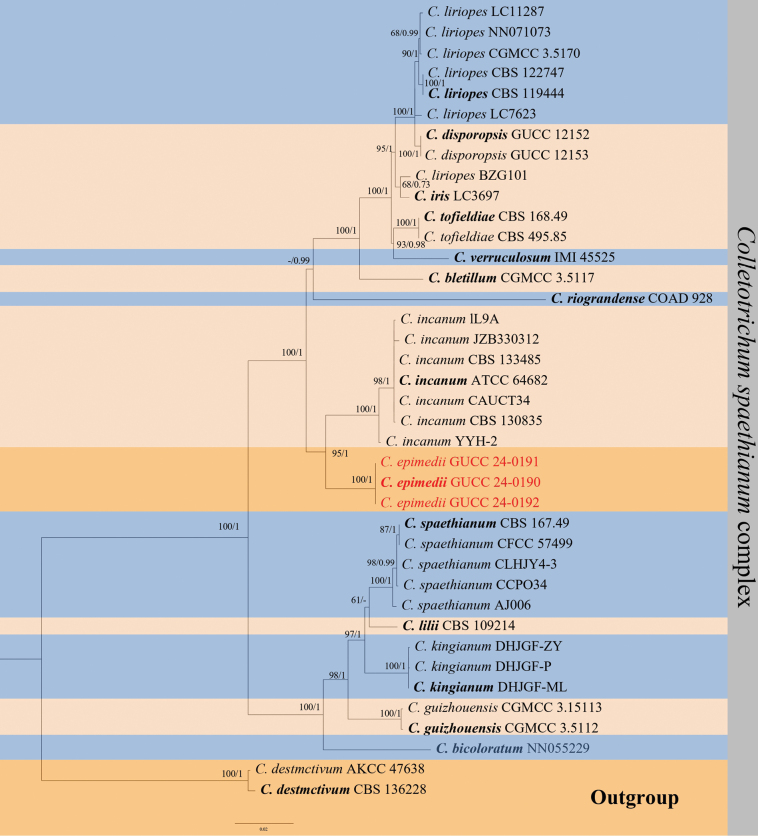
Phylogenetic tree of the *Colletotrichumspaethianum* complex based on multi-gene sequences (ITS, *act*, *tub2*, *gapdh*, *chs-1* and *his3*). Support values at the nodes indicate a maximum likelihood (ML) of > 60% and Bayesian posterior probability (BYPP) of > 0.70. The outgroup is *C.destructivum* CBS 136228 and *C.destructivum* AKCC 47638. The strains used were GUCC 24-0190, GUCC 24-0191 and GUCC 24-0192. The scale bar represents 0.02.

**Figure 4.**
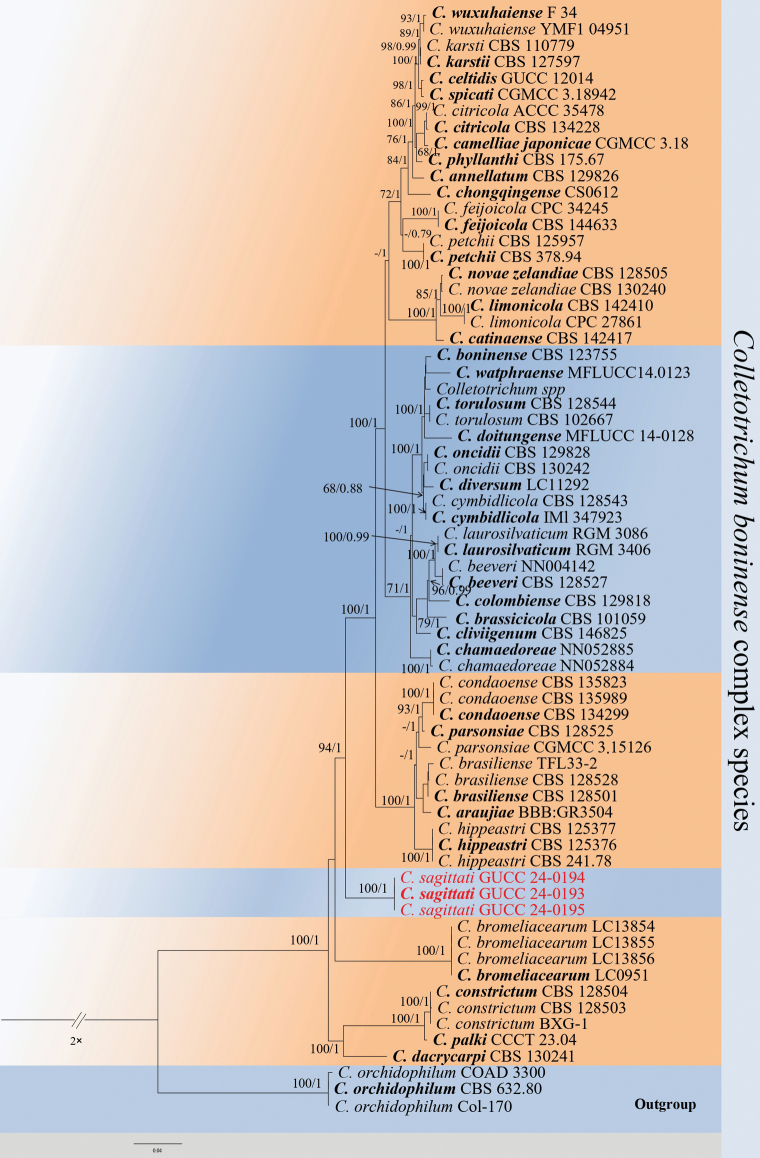
Phylogenetic tree of the *Colletotrichumboninense* complex constructed using multi-gene sequences (ITS, *act*, *tub2*, *gapdh*, *chs-1*, *his3* and *cal*). Support values at the nodes indicate a maximum likelihood (ML) of > 60% and Bayesian posterior probability (BYPP) of > 0.70. The outgroup is *C.euphorbiae* CBS 134752, COAD 3300, and Col-170. The strains used are GUCC 24-0193, GUCC 24-0194 and GUCC 24-0195, with a scale bar of 0.04.

In the phylogenetic tree (Figs 3, 4), the isolates from this study formed two distinct, well-supported clades and, thus, were considered to represent two previously unknown species. *C.epimedii*GUCC 24-0190, GUCC 24-0191 and GUCC 24-0192 without the DNA base differences in six loci amongst strains (ITS, *gapdh*, act, *his3*, *chs-1* and *tub2*) form an independent branch with strong support (ML = 95, PP = 1) sister to *C.incanum* (Fig. 3). Similarly, in the phylogenetic tree (Fig. 4), *C.sagittati*GUCC 24-0193, GUCC 24-0194 and GUCC 24-0195 also form an independent branch with strong support (ML = 94, PP = 1).

### ﻿Taxonomy

#### ﻿ Colletotrichum epimedii

Taxon classificationFungiGlomerellalesGlomerellaceae

K.Y. Jiang & Zhong Li sp. nov.

8B2F7AB5-613C-5A63-91B4-67DA2E4ED84E

 856528

Fig. 5

##### Etymology.

Named after the host plant genus, *Epimedium*.

##### Type.

China • Guizhou Province, Kaili City, Bibao Town (26°30'38"N, 107°37'23"E), from leaves of *E.sagittatum*, Apr 12, 2024, KY Jiang (holotype HGUP 21489, ex--holotype culture GUCC 24-0190).

**Figure 5.**
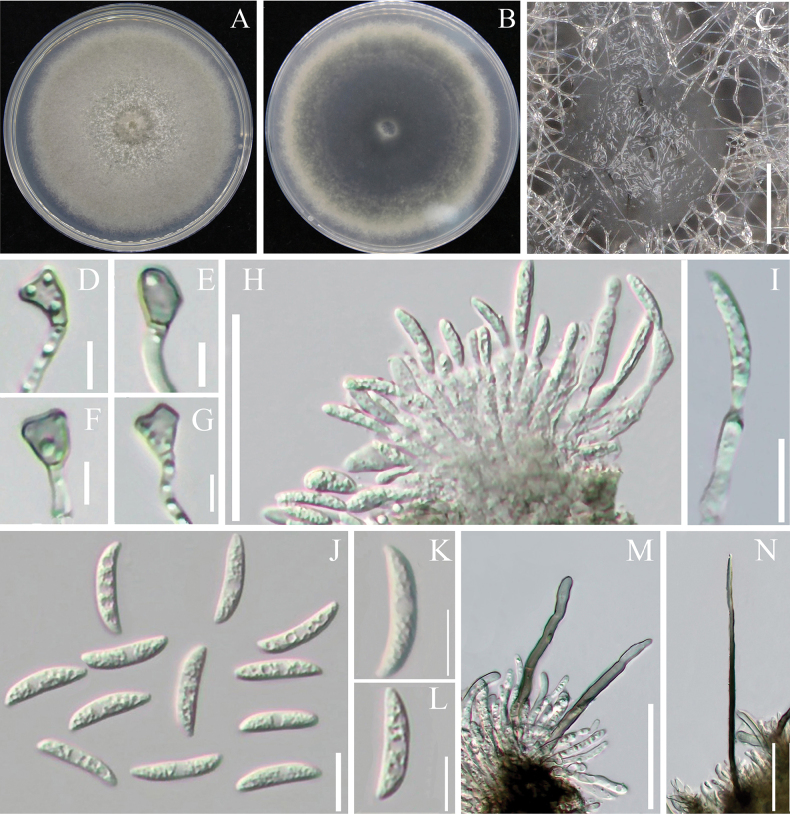
Morphological characteristics of *Colletotrichumepimedii***A** upper surface of the colony **B** underside of the colony **C***Conidiomata***D–G** appressoria **H, I** conidiophores **J–L** conidia **M, N** setae. Scale bars: 100 μm (**C**); 5 μm (**D–J**); 10 μm (**I, J–L**); 50 μm (**H**); 50 μm (**M, N**).

##### Description.

**Sexual morph**: Not observed. Asexual morph: ***Conidiomata***, globose to irregular, ash black. ***Setae*** and ***conidiophores*** formed on a cushion of dark brown and are non-branched. ***Setae*** medium to dark brown, straight, 81.2–168.5 μm long, 1–2 septate, tip acut. ***Conidiophores*** hyaline, unbranched, upon maturation of the ***conidia***, the apical portion undergoes constriction to form an ampulla or bowling pin-shaped structure, followed by subsequent detachment of the developed conidium. ***Conidia*** rough, non-septate, crescent or slightly curved in shape, with a near 1/2 mid-section having a depressed shape or multiple depressions, more towards the round or somewhat acute apex, base truncate, 16.5–18.8 × 4.3–5.4 μm (mean ± SD = 17.9 ± 0.8 × 4.7 ± 0.3 µm, L/W = 3.9). ***Appressoria*** single, grey–brown, irregularly shaped, 5.0–8.2 × 3.4–5.4 μm (mean ± SD = 6.1 ± 1.0 × 4.4 ± 0.4 µm, L/W = 1.4).

##### Culture characteristics.

Colonies on PDA taupe, rapidly growing to 8 cm within 7 days at 28 °C, with a dense mycelium, covered by a velvety grey–brown aerial mycelium on the surface. The reverse side of the colony is black in the centre, gradually lightening towards the edge and fading to grey.

##### Notes.

Multi-locus phylogenetic analysis indicates that the three *C.epimedii* strains form distinct branches; our taxonomic unit *C.epimedii* belongs to the *Spaethianum* complex. It shares low sequence similarity with the phylogenetically related species *C.incanum* at *act* (96%), *chs-1* (98%), *gapdh* (92%), *his3* (94%), *tub2* (98%) and ITS (99%). Morphologically, *C.epimedii* and *C.incanum* had different colony characteristics on PDA. The *C.incanum* colony has fewer mycelia, growing closely against the plate, whereas *C.epimedii* has a dense mycelium. Both strains are dark brown but had different conidia sizes: *C.epimedii* had shorter but wider conidia than *C.incanum*, length (16.5–18.8 μm vs. 17.0–21.9 μm), width (4.3–5.4 μm vs. 2.3–3.7 μm) and L/W ratio (3.9 vs. 6.5). The setae of *C.epimedii* were also slightly shorter than those of *C.incanum* (81.2–168.4 μm vs. 74–202 μm) (Yang et al. 2014). Considering both molecular phylogenetics and morphological characteristics, *C.epimedii* was identified as a new species.

#### ﻿ Colletotrichum sagittati

Taxon classificationFungiGlomerellalesGlomerellaceae

K.Y. Jiang & Zhong Li sp. nov.

97A10158-2251-5945-B3A7-CDE28D5E370C

 856529

Fig. 6

##### Etymology.

Named after the host plant species *sagittatum*.

##### Type.

China • Guizhou Province, Kaili City, Bibao Town (26°30'38"N, 107°37'23"E), from leaves of *E.sagittatum*. 12 Nov, 2024, KY Jiang (holotype HGUP 21490, ex-holotype culture GUCC 24-0193).

**Figure 6.**
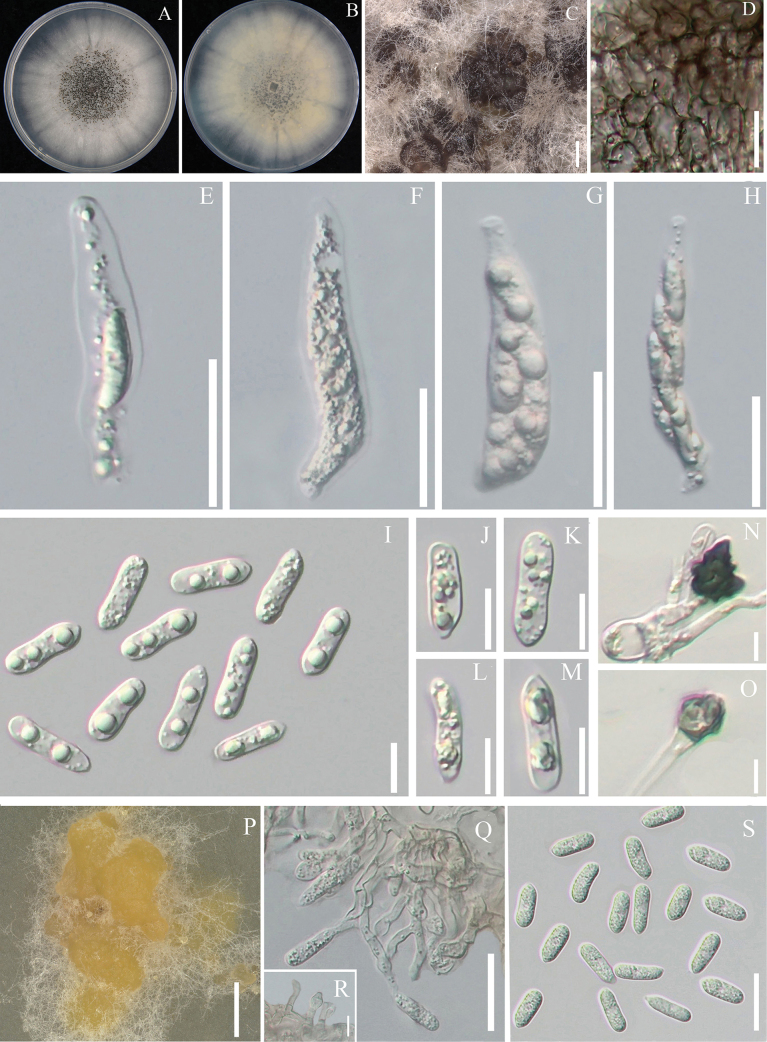
Morphological characteristics of *Colletotrichumsagittati***A** colony surface **B** colony reverse **C** ascomata **D** surface of the ascomata **E–H** asci **I–M** ascospores **N, O** appressoria **P** conidiomata **Q, R** conidiophores and Conidiogenous cells **S** conidia. Scale bars: 2.5 cm (**C**); 25 μm (**D–H, Q**); 10 μm (**I–M, R**); 25 μm (**P**); 5 μm (**N, O**); 20 μm (**S**).

##### Description.

**Asexual morph: *Conidiomata***, irregular, orange. ***Setae*** not observed. ***Conidiophores***, formed directly on hyphae, usually reduced to conidiogenous cells, laterally. ***Conidia*** hyaline, smooth-walled, aseptate, straight, few conidia slightly curved, cylindrical, the apex and base rounded 14.6–17.9 × 4.9–6.8 μm (mean ± SD = 16.0 ± 0.9 × 6.2 ± 0.6 µm, L/W = 2.56). ***Appressoria*** single, dark brown, irregularly, a small amount. **Sexual morph**: ***Ascomata*** perithecia, clustered, superficial, spherical, medium to dark brown, covered with sparse ***Asci*** unitunicate, 8–spored, cylindrical or rod-shaped, smooth-surfaced and slightly pointed at the apex, 38.7–70.5 × 11.0–15.7 μm. ***Ascospores*** single or multiseriately arranged, aseptate, hyaline, smooth-walled, cylindrical, blunt rounded ends or slightly protruding at one end, 16.0–19.4 × 3.7–5.6 μm (mean ± SD = 17.1 ± 1.0 × 4.8 ± 0.4 μm and L/W ratio = 3.6).

##### Culture characteristics.

Colonies on PDA flat, with poorly developed aerial mycelium, closely adhered to the medium surface, with numerous ascomata in the centre. Mycelium white, reverse same colour, growth 7 cm in 14 d.

##### Notes.

Multi-locus phylogenetic analysis indicates that the three strains of *C.sagittati* formed a distinct branch within the *C.boninense* species complex (Fig. 4). Every locus sequenced for these species differed from currently recognised *Colletotrichum* species. A BLASTn search of *C.epimedii* sequences in the NCBI GenBank revealed low similarity to other species. The highest similarities for *cal*, *act*, *chs-1*, *GADPH*, *his3*, ITS and *tub2* were found with *C.hippeastri* CSSG1 (92.01%), *C.karsti* AGMy0178 (92.54%), *C.chamaedorea*LC13867 (98.34%), *C.bromeliacearum*LC13855 (77.78%), *C.liriopes* HZ-1 (91.30%), *C.boninense* INBio-275813 (97.96%) and *C.karsti* BRIP (91.76%). In morphology, they can be distinguished from *Colletotrichumhippeastri* by its smaller conidia (14.6–17.9 × 4.9–6.8 vs. 19–37.5 × 5.5–8.5) (Damm et al. 2012). Additionally, *C.sagittati* produces greater *conidia* than *Colletotrichumbromeliacearum* (14.6–17.9 × 4.9–6.8 vs. 8.5–16 × 5–7.5) (Liu et al. 2022). Based on the integrated molecular phylogenetics and morphology, *C.sagittati* was identified as a new species.

## ﻿Discussion

In fungal identification, the integrated application of morphological and molecular biological approaches represents the most widely utilised metho­dology and demonstrates enhanced taxonomic efficacy. (Cai et al. 2009; Jayawardena et al. 2016). The genus *Colletotrichum*, commonly known as anthracnose fungi, is an important group of plant pathogens that can infect more than 3,200 plant species, causing substantial harm to various economic crops worldwide. This genus is characterised by its ubiquity and severity (Cannon et al. 2012; Guarnaccia et al. 2017; Fu et al. 2019). The classification of *Colletotrichum* has historically been complex, but the application of multi-locus molecular methods has facilitated the identification and categorisation of numerous *Colletotrichum* species into distinct species complexes (Cannon et al. 2012; Crouch 2014; Damm et al. 2019). Currently, the genus is divided into 16 complexes, with more than 750 new species described based on different host plants (Liu et al. 2022). Many closely related species are difficult to differentiate based solely on morphological characteristics. Therefore, constructing multi-gene phylogenetic trees in conjunction with morphological features is fundamental for identifying species within this genus and serves as a primary basis for describing new species (Cai et al. 2009; Jayawardena et al. 2016). Utilising combined multi-gene sequences from ITS, *gapdh*, *chs-1*, *his3*, *act*, *tub2*, and *cal* yields better results than single-gene analyses. This study constructed a phylogenetic tree based on combined multi-gene sequences of ITS, *gapdh*, *chs-1*, *his3*, *act*, *tub2*, and *cal*. Pathogenicity tests led to the identification of two new *Colletotrichum* species associated with anthracnose disease on *E.sagittatum*, named *C.epimedii* and *C.sagittati*.

In recent years, the cultivation area of *Epimedium* has been increasing to meet growing market demand. However, this trend has also led to issues such as a high incidence of diseases and the rapid spread of diseases within plantations. Therefore, accurate diagnosis and prevention of these diseases are crucial. The current reports of anthracnose on *E.sagittatum* are limited to two cases caused by *C.fructicola* and *C.karstii* (Hou et al. 2024b; Lin et al. 2025). The symptoms of anthracnose observed here are similar to those reported by Hou et al. (2024b). However, the involved pathogens and their respective species complexes differ. Although we isolated *C.fructicola* from *E.sagittatum*, its pathogenicity was weak. Moreover, this variation in pathogenicity among regions may be due to regional adaptation of the pathogens. Thus, we hypothesise that anthracnose in *E.sagittatum* may represent a complex disease. Before implementing effective control and prevention strategies, it is essential to accurately identify and understand the types of pathogens involved. Further identification and classification of the pathogens responsible for anthracnose are warranted.

## ﻿Conclusion

This study identified two novel species of anthracnose fungi, *C.epimedii* and *C.sagittati*, responsible for anthracnose in *E.sagittatum*. These species belong to the *C.spaethianum* and *C.boninense* complexes. To effectively control the disease, further research is required to elucidate how these two strains respond to climatic conditions, common fungicides and prevalent *Epimedium* genotypes. Such studies will aid in developing more targeted disease management strategies.

## Supplementary Material

XML Treatment for Colletotrichum epimedii

XML Treatment for Colletotrichum sagittati

## ﻿Additional information

### Data availability

All of the data that support the findings of this study are available in the main text.
